# Prevalence of transfusion transmitted virus (TTV) genotypes among HCC patients in Qaluobia governorate

**DOI:** 10.1186/1743-422X-4-135

**Published:** 2007-12-06

**Authors:** Mohamed M Hafez, Sabry M Shaarawy, Amr A Hassan, Rabab F Salim, Fatma M Abd El Salam, Amal E Ali

**Affiliations:** 1 Virology and Immunology Unit, Cancer Biology Department, National Cancer Institute, Cairo University, 1st Kasr El-Aini st, Cairo, Egypt; 2 Biochemistry department Benha Faculty of medicine Benha University, Banha, Egypt

## Abstract

**Background:**

Transfusion Transmitted virus (TTV) is a novel single-stranded DNA virus that was identified in patients with post-transfusion hepatitis of non-A-G type. Clinical significance of TTV infection was analyzed in Egyptian hepatocellular carcinoma (HCC) patients. The present study attempted to clarify these issues in Egypt, particularly in Qaluobia governorate, a country known for its high endemicity of liver disease and hepatotropic viruses.

**Methods:**

TTV are determined in the serum of 60 samples obtained from HCC and liver cirrhosis (LC) patients and 30 healthy individuals. TTV DNA is amplified by nested-PCR with TTV-specific mixed primers derived from the conserved open reading frame 1 (ORF1) region followed by digestion with restriction enzyme. Using the enzymes *Hae*III, *Dra*I, *Eco*RI and *Pst*I, we are able to distinguish between the four TTV genotypes.

**Results:**

The positive rate of TTV detection was 46.7%, 40% and 36.7% among HCC, LC patients and healthy individuals respectively. The more prevalence genotype was detected in the positive serum samples was genotype 1 (35.7%) in HCC patients, (50%) in LC and (63.3%) in healthy individuals, Genotype 5 (21.4%), (25.5%) and (18.2%) in HCC, LC and healthy individuals respectively.

**Discussion:**

This study indicates that TTV is commonly present in adult patients with HCC and LC as well as healthy individuals. The most prevalence TTV genotype is genotype 1. It seems that the infection neither contribute to the severity of liver disease no to the causation of HCC.

## Background

Hepatocellular carcinoma (HCC) is the fifth most common cancer but the third leading cause of cancer death in the world. The major etiology of HCC/liver cancer in people is hepatitis B virus (HBV), followed by hepatitis C virus infection (HCV). A small single-stranded DNA virus, named TT virus (TTV), was discovered in Japan from patients with non-A-G transfusion-acquired hepatitis [1]. Knowledge about novel hepatotropic virus TTV is growing fast, but some fundamental aspects remain to be elucidated. Its prevalence and clinical significance are being assessed worldwide, however its relationship with aggravation and progression to severe liver disease and HCC remain controversial. TTV DNA has been detected in many healthy and the diversity of the strains of the virus has been reported [2]. TTV DNA was detected in 12% of healthy blood donors, although the serological prevalence of TTV infection in healthy blood donors was lower than that in patients with fulminant or chronic cryptogenic liver diseases [3]. TTV infection was also investigated [4-7] in patients on maintenance hemodialysis (HD), as they are assumed to be at risk of blood-borne virus infections such as hepatitis C virus (HCV), because of the repeated blood transfusion and the high frequency of exposure to invasive techniques [8,9].

Analyses based on a phylogenetic tree constructed using the open reading frame (ORF) 1 sequence of TTV, showed that the virus could be classified into different genotypes. At least four groups comprising 23 genotypes of TTV have been identified [10,11]. In addition four new genotypes have recently been identified and classified as TTV group 5 [12].

In Africa, HCV is reported to have very high prevalence in many countries with the unique genotypes 4 and 5 [13]. In Egypt, where very high prevalence was reported in both risky [14] and healthy [15] groups mostly with genotype 4 [16]. Moreover, in Egypt previous exposure to parenteral antischistosomal therapy was considered as one of the most important iatrogenic causes of HCV spread there [17], and an unanswered question exists about how far such an exposure affected transmission of other blood-borne and newly discovered viruses like TTV in this unique community.

The aim of the present study was to assess the prevalence of TTV infection among Egyptian patients with LC and HCC, and to detect its genotypes. The present study attempted to clarify these issues in Egypt, particularly in Qaluobia governorate, a country known for its high endemicity of liver disease and hepatotropic viruses. Also to assess the impact of this virus on the severity of liver disease and its association with the development of HCC.

## Materials and methods

The study was approved by ethical committee and informed consents were obtained from all parents of each patients participating in the study. This study included ninety cases, 30 patients with HCC and 30 patients with LC; they were selected from outpatient's clinic and in-patients of Benha university hospital. Both cases and control were subjected to full history, clinical examination and investigations required for proper clinical diagnosis. Serum samples were collected from patients and healthy individual in a period from December 2004 to January 2006 and stored at -20°C until used. The mean age of the patients ranged is 60.3, 53.2 and 38.7 years for HCC, LC and healthy individuals respectively.

### Virological investigations

Patients and control were examined for HBsAg (Adalits-Italy) and HCV-Ab (INNOGENATICS N.V. Belgium) according to the manuscript.

### Determination of TTV by PCR

#### 1 – DNA extraction

The QIAamp DNA extraction kit (QIAGEN GmbH, Hilden Germany) was employed for DNA extraction from serum samples according to the manufacturer's instructions.

**Figure 1:**
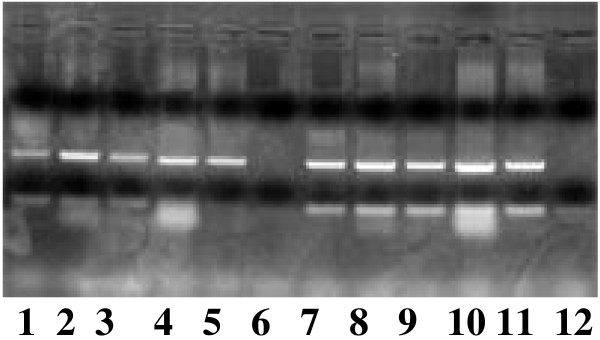
Ethidium bromide stained gel electrophoresis of TTV-PCR product showing positive (lane 1,2,3,4,5,78,9,10 and 11) and negative (lane 6, and 12) signals.

TTV sequences were amplified in hemi-nested PCR using primers NG059, NG061, and NG063. The first round PCR was carried out in 35 cycles consistent of 9 min at 96°C, followed by 35 cycles consisting of denaturation for 30 s at 94°C, annealing for 45 s at 60°C, and extension for 45 s at 72°C, with the sense primers 5'ACAGACAGAGGAGAAGGCAACATG3' (nt 1920–1943, NG059) and anti-sense primer 5'CTGGCATTTTACCATTTCCAAAGTT3' (nt 2205–2180, NG063). The second round of PCR was performed with the sense primer 5'GGCAACATGTTATGGATAGACTGG3' (nt 1935–1958, NG061) and the anti-sense primer NG063 for 25 cycles, under the same conditions as used for the first round of PCR. In each PCR assay, one negative and two positive controls were tested together with the serum samples. The amplification products 271 bp were visualized on an ethidium bromide-stained 2% agarose gel figure 1.

#### RFLP analysis

**Figure 2:**
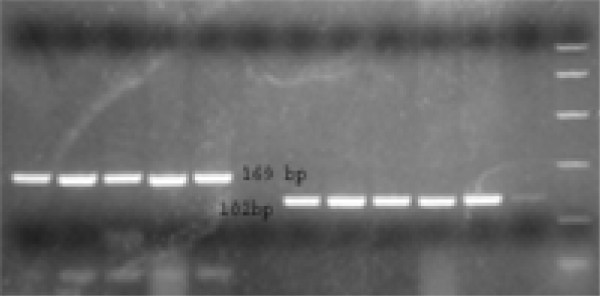
Ethidium bromide stained gel electrophoresis of TTV-PCR product after digestion with restriction enzyme.

A new genotyping assay, based on RFLP analysis, was developed. The alignment of sequences determined as above revealed the presence of genotype-specific restriction sites, combinations of which determined each genotype as shown in figure 2. Restriction digestions were carried out with 10 ul of the second round PCR products for 15 min after adjustment with 10 U enzyme reaction buffer according to the manufacturer's instructions. Reactions were carried out with 10 units of NdeI, PstI and Hin1II (Fermentas, USA) at 37°C. The digested PCR products were electrophoresed on 3% agarose gels, stained with ethidium bromide. The RFLP pattern was then evaluated under ultraviolet light.

#### Statistical analysis

For categorical variables differences between groups were analyzed by using analysis of student t test for two groups' comparison and chi square test for non parametric values.

## Results

The present study is conducted on sixty patients classified into two groups: group one includes thirty patients with hepatocellular carcinoma (HCC) (25 males and 5 females) and the second group includes thirty patients with liver cirrhosis (LC) (19 males and 11 females). They selected from inpatients and outpatients' clinic of Benha university hospital, and thirty healthy volunteers (18 males and 12 females) were collected from blood bank and considered as control group. All Characteristic features of the different studied groups are shown in table 1.

**Table 1:** Characteristic features of the studied groups:

	HCC	LC	Control
	N = 30	N = 30	N = 30

Age	60.3 ± 6.9	53.2 ± 5.5	38.7 ± 11.03
Sex	♂ 25/30 (83.3%)	♂ 19/30 (63.3%)	♂ 17/30 (56.7%)
	♀ 5/30 (16.7%)	♀ 11/30 (36.7%)	♀ 13/30 (43.3%)
Blood transfusion	14/30 (46.7%)	12/30 (40%)	3/30 (10%)
Alcohol intake	4/30 (13.3%)	3/30 (10%)	0/30 (0%)

TTV DNA was found in 26 out of the 60 subjects included in this study (43.3%). Mean ages were similar in TTV-infected and uninfected subjects of the HCC, LC and the control group. There is a significant different was observed between male and female in different studies groups in HCC 11/25(44%) in male, 3/5(60%) in female whereas in LC 5/19(26.4%) male was infected compared to 7/11(63.6%) in female whereas in control group 7/17(41%) male and 7/11(63.6%) female was infected.

In HCC patients with blood transfusion, 75.1% had TTV infection. In LC patients with blood transfusion 50% had TTV infection

Although a higher prevalence of circulating TTV DNA was detected in HCC patients 46.7%(14/30) in which 42.9% had HCV, 7.1% had HBV infection, non had both HCV and HBV and (11/30)36.7% in healthy blood donors in which 4(36.4%) had HCV, the differences between groups were not statistically significant. In the LC patients, TTV DNA was found in twelve out of 30 LC patients in which 8(41.7%) had HCV, 1 (7%) had HBV, 2(16.7%) had both HCV and HBV table 2. No statistical significant difference in TTV prevalence was observed between HCC patients and LC patients with/without co-existing HCV or HBV infection.

**Table 2:** Virological data of HCC and LC patients in relation to TTV DNA viraemia.

	HCC		LC		
	
	+ve TTV n = 14	-ve TTV n = 16	+ve TTV n = 12	-ve TTV n = 18	Total n = 60
HCV Positive	6/14(42.9%)	8/16(43.8%)	8/12 (41.7%)	9/18 (50%)	26/60 (43.3%)
HBV positive	1/14(7.1%)	3/16(18.8%)	1/12 (7%)	2/18 (11%)	7/60 (11.7%)
Both HCV & HBV positive	1/14 (7.1%)	2/16(12.5%)	2/12 (16.7%)	2/18 (11%)	7/60 (11.7%)
Both HCV & HBV negative	7/14 (50%)	4/16 (25%)	3/12 (33.3%)	6/18(33.3%)	20/60 (3.3%)

P value	> 0.05				

There were no statistically significant differences between TTV-infected and non infected patients in relation to virological features of HCV and HBV.

### Distribution of TTV genotypes among different study group

The highest TTV genotypes in the HCC group were genotype 1 and 5 which represent 4/14 (28.6%) and 3/14 (21.5%), whereas in LC the genotype1 was 6/12 (50%) and 3/12 (25%) genotype 5 whereas among control group genotype 1 was 7/11(63.6%) and 2/12 (16.6%) genotype 5. There is no significant different was observed among different study group in relation to TTV genotype.

There is no significant different was observed among different study group in relation to TTV genotype and HCV and/or HBV coinfection. In HCC patient, TTV-coinfected with HCV 2/4 (50%) is genotype 1 and 2/3 (66.7%) genotype 5. In LC patient, TTV-coinfected with HCV 4/6 (66.7%) is genotype 1 and 3/3 (100%) genotype 5. Three out of 7 (42%) genotype 1 was observed among control group whereas 1/2 (50%) genotype 5.

## Discussion

TTV was first detected in subjects with post-transfusion hepatitis and indicated as a possible an etiologic agent of non A-non C hepatitis [1]. Although TTV DNA was found at high concentrations in liver tissue and in serum of patients with liver disease [3], several studies also reported a high endemicity of infection in subjects with no evidence of hepatitis [18]. Therefore, the role of TTV as a cause of liver disease is controversial. In this study, we have evaluated the prevalence of serum TTV DNA and their genotypes in relation to liver disease in two examples HCC and LC patients as well as in volunteer blood donors as control.

In this study, nucleic acids were extracted from a convenience sample comprising 60 serum samples collected from HCC and LC patients and 30 control groups. We detected an overall prevalence of TTV infection of 43.3%, which is higher than previously reported prevalence in patients with liver disease, in volunteer or commercial blood donors and in high risk populations from Western countries (1–13%) [18]. By contrast, our data are similar to those reported in patients with chronic hepatitis or cirrhosis in Japan, Taiwan and Thailand [3,19].

The use of a seminested PCR protocol gave estimates for the prevalence of TTV infection of 46.7%, 36.7% and 41%. This value is lower those reported for Turkish (75%) [20], Polish (78%) [21], Thailand (62%), Korea (53%) and among nationals and non-nationals in United Arab Emirates (40% and 89% respectively) and in a group of 137 Japanese subjects with no reported liver disease (70%) [22-25]. Interestingly, in the present study, the rate of TTV infection did not significantly differ between patients with liver disease, with or without HCV infection, and healthy blood donors. The high prevalence of TTV in general population, may complicate linking TTV to hepatic disease and other pathologic states [26]. This unusual feature among viruses aroused the proposal that TTV might be a commensal virus or part of human microflora [27]. Another major complication is the extreme heterogeneity of TTV genome, its divergent genogroups [1-5], and genotypes [28] each of which possess distinct biologic properties and pathogenic potentials [12,26].

A study even describes an overall prevalence of 94% in a sample representative of the general population [29]. Such differences may be explained by the differences in population sampling or in the choice of primers [18]. The rate of TTV infection detected in blood donors in the present study (36.7%) is considerably higher compared to the results of other studies on Italian donors reporting prevalences ranging from 18.6% to 22% [29,30], but within the range reported by a French study [31]. This could be due to the different populations analyzed.

TTV is characterized by an unusually high degree of sequence variability compared to other DNA viruses and several distinct TTV genotypes have been described [3,19,28]. Genotypes 1, 2, 3 and 4 appear to be widely distributed throughout the world, whereas the prevalence of other putative TTV genotypes has not been fully assessed and might be geographically restricted [31-35].

Characterization of the genotypes of TTV circulating in our study population was carried out by restriction fragment length polymorphism (RFLP). Our analysis reveals the existence of six different genotypes of TTV and G1 shows the highest distribution among patients in Qaluobia governorate. The RFLP of all TTV-DNA positive samples revealed that prevalent genotypes 1 was the most frequently found 17/36 (47%), while 8/36 (22%) showed genotypes 5. A similar epidemiological profile for TTV genotypes has also been described for Italian subjects [30,36].

In regard to TTV, the prevalence of G1 and G2 is very high worldwide, and these are probably major genotypes of TTV. The distribution of the major TTV genotypes, G1 and G2, was not related to their geographic distribution. This suggests that TTV, a single-stranded DNA virus, probably spread all over the world a long time ago and coexisted with humans for long without pathogenicity. Our data is in accordance with others that G1 was the most common genotype of TTV in Japan; however, it is still unclear whether any correlation exists between the TTV genotypes and their geographical distribution or pathogenicity. Genotype 6 has the lowest found, similarly Genotype 6 has rarely found outside Japan, even in large surveys including patients from different parts of the world [19] and its presence in Italy has been inferred by *Maggi*, [37], on the basis of RFLP analysis.

As the distribution of the different TTV genotypes might have potentially important clinical and epidemiological implications, it is necessary to evaluate the association of particular genotypes of TTV with the severity of liver diseases. However, our results show that no significant association could be identified between TTV genotypes and either LC or HCC.

The pathogenic implications of TTV genomic heterogeneity are unknown. Available data showing low disease associations of TTV infection are derived from studies that considered mostly patients infected with genotype 1. The possibility that TTV genotypes may differ in their pathogenic potential cannot be excluded, since the prevalence and clinical correlations of uncommon TTV genotypes have not been explored so far. Analysis of each TTV genotype revealed that co-infection of TTV genotype 5 with HCV is more frequent 66.7% in HCC, 100% in LC and 50% in control group, comparing to TTV genotype1 50%, 66.7% and 42% respectively. The multiple TTV-genotype co-infections was not found, others did not find any relationship between liver diseases and TTV genotypes and reported that TTV has several different genotypes as does HCV, and so there probably are specific TTV genotypes causing severe liver diseases or other diseases, although it still remains unclear whether TTV is a direct cause of disease or not [19,28].
